# Effects of Serine or Threonine in the Active Site of Typical 2-Cys Prx on Hyperoxidation Susceptibility and on Chaperone Activity

**DOI:** 10.3390/antiox10071032

**Published:** 2021-06-25

**Authors:** Carlos A. Tairum, Melina Cardoso Santos, Carlos Alexandre Breyer, Ana Laura Pires de Oliveira, Vitoria Isabela Montanhero Cabrera, Guilherme Toledo-Silva, Gustavo Maruyama Mori, Marcos Hikari Toyama, Luis Eduardo Soares Netto, Marcos Antonio de Oliveira

**Affiliations:** 1 Instituto de Biociências, Universidade Estadual Paulista, UNESP, São Vicente 01049-010, Brazil; tairumjr@usp.br (C.A.T.); melina.css@hotmail.com (M.C.S.); carlosbreyer@gmail.com (C.A.B.); alp.oliveira@unesp.br (A.L.P.d.O.); vitoria.isabela.montanhero@gmail.com (V.I.M.C.); marcos.toyama@unesp.br (M.H.T.); 2 Departamento de Genética e Biologia Evolutiva, Instituto de Biociências, Universidade de São Paulo, São Paulo 01049-010, Brazil; 3 Laboratório de Biomarcadores de Contaminação Aquática e Imunoquímica, Departamento de Bioquímica, Universidade Federal de Santa Catarina, Florianópolis 88040-900, Brazil; guilherme.toledo@ufsc.br; 4 Laboratório de Ecologia Molecular, Instituto de Biociências, Universidade Estadual Paulista, UNESP, São Vicente 01049-010, Brazil; gustavo.mori@unesp.br

**Keywords:** 2-Cys Prx, catalytic triad, organic hydroperoxides, oligomerization, hyperoxidation, chaperone

## Abstract

Typical 2-Cys peroxiredoxins (2-Cys Prx) are ubiquitous Cys-based peroxidases, which are stable as decamers in the reduced state, and may dissociate into dimers upon disulfide bond formation. A peroxidatic Cys (C_P_) takes part of a catalytic triad, together with a Thr/Ser and an Arg. Previously, we described that the presence of Ser (instead of Thr) in the active site stabilizes yeast 2-Cys Prx as decamers. Here, we compared the hyperoxidation susceptibilities of yeast 2-Cys Prx. Notably, 2-Cys Prx containing Ser (named here Ser-Prx) were more resistant to hyperoxidation than enzymes containing Thr (Thr-Prx). In silico analysis revealed that Thr-Prx are more frequent in all domains of life, while Ser-Prx are more abundant in bacteria. As yeast 2-Cys Prx, bacterial Ser-Prx are more stable as decamers than Thr-Prx. However, bacterial Ser-Prx were only slightly more resistant to hyperoxidation than Thr-Prx. Furthermore, in all cases, organic hydroperoxide inhibited more the peroxidase activities of 2-Cys Prx than hydrogen peroxide. Moreover, bacterial Ser-Prx displayed increased thermal resistance and chaperone activity, which may be related with its enhanced stability as decamers compared to Thr-Prx. Therefore, the single substitution of Thr by Ser in the catalytic triad results in profound biochemical and structural differences in 2-Cys Prx.

## 1. Introduction

Typical 2-Cys peroxiredoxins (2-Cys Prx) of the AhpC/Prx1 sub-family are peroxidases widely distributed in prokaryotes and eukaryotes that reduce hydroperoxides with high efficiency [1,2,3,4]. These enzymes are very abundant, representing 0.1–1.0% of all soluble proteins in the cells [5,6,7,8] and capable of decomposing distinct hydroperoxides [9,10]. Additionally, typical 2-Cys Prx are mediators of redox signaling and can act as molecular chaperones [11,12,13,14,15].

The 2-Cys Prx use a fully conserved N-terminal cysteine residue (peroxidatic cysteine-C_P_), which is responsible for the nucleophilic attack on the hydroperoxide and then becomes oxidized into a sulfenic acid (C_P_-SOH), which can condensate with a second Cys residue (resolving cysteine, C_R_-SH) forming an intermolecular disulfide (C_P_-S-S-C_R_). In most cases, these disulfide bonds are reduced by thioredoxin (Trx) [16,17,18,19]. The high reactivity of the C_P_ is related to the microenvironment in which it is found. C_P_ is in close proximity to other two fully conserved residues (Thr/Ser and Arg), forming polar interactions that increase the electrophilicity of H_2_O_2_, and facilitate the nucleophilic attack by the thiolate on C_P_ [20]. As expected, C_P_ [21,22,23], as well as Thr/Ser and Arg [3,23,24,25], are essential for the efficient reduction of hydroperoxides. Thus, these three residues (C_P_, Thr/Ser, and Arg) compose the so-called catalytic triad [3,22,26].

Under high hydroperoxide concentrations, C_P_ can undergo further oxidation generating hyperoxidized species (C_P_-SO_2_H or C_P_-SO_3_H) rather than forming the disulfide bond, inactivating Prx turnover by Trx [27,28]. However, the enzyme sulfiredoxin (Srx) can reduce C_P_-SO_2_H to C_P_-SOH in a process dependent on ATP hydrolysis for typical 2-Cys Prx [27,28]. Hyperoxidized 2-Cys Prx are frequently associated with a molecular chaperone (holdase) function and may impact signaling pathways, like cell growth and circadian rhythm [29,30,31,32]. In addition to C_P_ hyperoxidation, high temperatures can also confer chaperone function to these enzymes [33,34,35,36].

**Figure 1:**
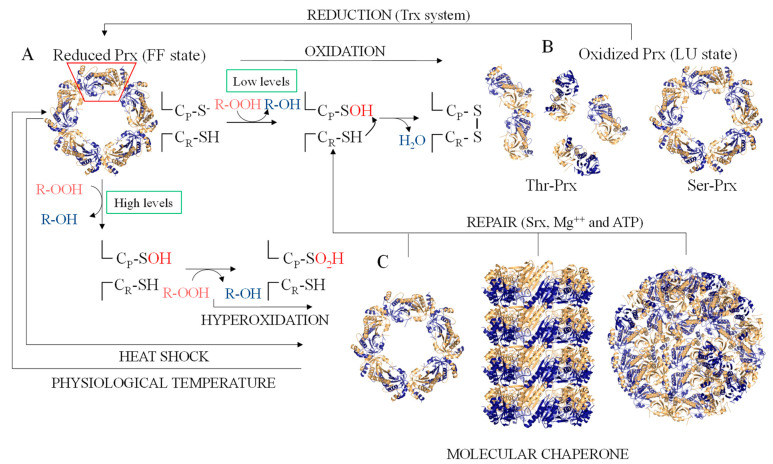
Structural and functional transitions of 2-Cys Prx. (**A**) In reduced (C_P_-S^−^) and in the fully folded (FF) state, 2-Cys Prx are decamers formed by association of five homodimers (α_2_[_5_]). The red trapeze denotes one of the homodimers that compose the decamer. The monomers of each homodimer are depicted in dark blue and light orange, represented in cartoon. At low hydroperoxides level, C_P_ is oxidized to sulfenic acid (C_P_-SOH) and then the condensation with C_R_ occurs, resulting in the formation of an intermolecular disulfide bond and locking the 2-Cys Prx in the locally unfolded (LU) state. (**B**) In 2-Cys Prx containing Thr (Thr-Prx), the disulfide formation results in decamer dissociation, while in 2-Cys Prx containing Ser (Ser-Prx), the decamers are stable even in the disulfide form. (**C**) At high levels of hydroperoxides, C_P_-SOH can react with another(s) hydroperoxide molecule(s) resulting in hyperoxidized species (C_P_-SO_2_H or C_P_-SO_3_H) and in the functional switch from peroxidase to chaperone. This functional switch may be accompanied by the appearance of species with molecular weight higher than the decamer. This process can be reversed by Srx, with ATP hydrolysis. The functional transition from peroxidase to chaperone can also be triggered by high temperatures and the peroxidase activity is restored at physiological temperatures. The 2-Cys Prx representations were made using the crystallographic coordinates of *Saccharomyces cerevisiae* enzyme (PDB = 3SBC).

A remarkable feature of 2-Cys Prx is the structural rearrangement that are triggered by C_P_ oxidation. Reduced 2-Cys Prx enzymes (C_P_-S^−^) are stabilized as decamers [(α_2_)_5_] and can assume the so called fully folded (FF) state (Figure 1A), in which C_P_ is located in the first turn of an α-helix near to N-terminal region of the protein and far away from C_R_ (10–16 Å), which is located in the C-terminus of these enzymes. Upon oxidation, partial unfolding of the α-helix containing C_P_-SOH (the so-called locally unfolded = LU state) allows its approximation to C_R_ with the consequent intermolecular disulfide bond formation [37]. In the disulfide form, the 2-Cys Prx decamers tend to dissociate into low molecular weight (LMW) species, mainly dimers [38,39,40] (Figure 1B). Moreover, when C_P_ is hyperoxidized or when subjected to heat shock, 2-Cys Prx can switch from decamers to higher molecular weight (HMW) species [32,33,35,41,42] (Figure 1C). Therefore, two reversible structural switches (FF–LU and decamers dissociation) are inter-connected and the stabilities of distinct conformers are influenced by thermal and/or redox factors.

The transitions and stability of distinct oligomers also rely in structural peculiarities of these enzymes. In the 2-Cys Prx from *Escherichia coli* (EcAhpC) mutations close to the C-terminal region precludes the decamer formation [43]. Factors as pH, ionic strength, and protein concentration can influence directly the quaternary state of 2-CysPrx, indicating that the oligomeric rearrangements are directly related to specialized functions of these enzymes in different organisms and cell compartments [39,44,45,46,47]. In that regard, we have shown that the presence of Thr or Ser in the catalytic triad influences the stabilities of distinct oligomeric states that 2-Cys Prx can assume [3]. Yeast Tsa1 and Tsa2 contain Thr or Ser in the active site, respectively. Both enzymes are decamers in the reduced state, but Tsa1 is mainly a dimer when oxidized and some are intermediate species (e.g., tetramers), while Tsa2 remains as decamer even after oxidation [3]. In addition, the mutation of catalytic Thr to Ser stabilizes Tsa1 in the decameric form even when oxidized [3]. The substitution of catalytic triad Thr to Ser also stabilizes typical 2-Cys Prx from *Salmonella typhimurium* and *Enterococcus faecalis* as decamers [48,49].

Here, we described that the presence of Thr and Ser in the active site also affected the sensibility of 2-Cys Prx to hyperoxidation. Previously, other structural features were described to confer sensibility of C_P_ to hyperoxidation, namely a GGLG motif (close to the active site) and a YF motif (found in a α-helix in the C-terminal portion) that are present almost uniquely in eukaryotic enzymes [40]. Posteriorly, the presence of the motif A (D-X_8_-N/G-X_10_-H-X_27_-S/G) and of the motif B (T-X_3_-S/T) were associated with increased resistance of 2-Cys Prx to hyperoxidation [50]. We show that yeast 2-Cys Prx (AhpC/Prx1 subfamily) with Ser in the catalytic triad (named here Ser-Prx) are more resistant to hyperoxidation than those containing Thr (named here Thr-Prx). Moreover, analysis of all the sequences of typical 2-Cys Prx present in the NCBI GenBank revealed that occurrence of Ser-Prx is rare in multicellular eukaryotes and more frequent in eubacteria. Therefore, we describe that bacterial Ser-Prx were also more stable as decamers and displayed more resistance to thermal inactivation than bacterial Thr-Prx. Furthermore, we found that cumene hydroperoxide (CHP) was more efficient than H_2_O_2_ to inactivate both Ser-Prx and Thr-Prx. Finally, we showed that bacterial Ser-Prx retained peroxidase activity and acquired molecular chaperone activity more readily than Thr-Prx after treatment at high temperatures.

## 2. Materials and Methods

### 2.1. Amplification, Cloning and Sequencing

Yeast wild type proteins (Tsa1, Tsa2, Trx1, and TrxR1) were obtained as previously described [3,51]. Bacterial AhpC and the corresponding thiol reductive systems were amplified by PCR from the genomic DNA of the following species/strains: *Escherichia coli*/ATCC25922, and *Pseudomonas aeruginosa*/ATCC29853. The *ahpc* gene from *Staphylococcus epidermidis*/ATCC12228 was synthesized by GenScript, based on the sequence SE_2357 from KEGG database, with optimized codons for expression in *E. coli*, and flanked with *Nde* I and *Bam* HI restriction sites. Amplified DNA fragments and synthetic gene were digested with *Nde* I and *Bam* HI and cloned into pET15b vector previously digested with the same enzymes. The resulting recombinant vectors were sequenced in an ABI 3730 DNA Analyzer (Applied Biosystems) using the Big Dye Terminator v3.1 Cycle Sequencing Kit (Applied Biosystems) and then were used to transform *E. coli* BL21 (*DE3*) or *E. coli* Tuner (*DE3*) strain (Sigma-Aldrich, Taufkirchen, Germany).

### 2.2. Site Directed Mutagenesis

The mutagenesis protocols were performed using Quick Change II Kit (Stratagene), following manufacturer’s instructions. The products were used to transform *E. coli* XL1-Blue strain (Agilent Technologies, Santa Clara, CA, USA) and single colonies were grown on Luria Bertani (LB) medium for 16 h. Then, plasmids were extracted, purified, and sequenced. Plasmids containing the expected single substitutions were used to transform *E. coli* BL21 (*DE3*) or *E. coli* BL21 Tuner (*DE3*) expression strains (Sigma-Aldrich). The BL21 (*DE3*) strain containing the plasmid pET15b/*tsa1*^t44s^ was previously obtained [3]. All strains used in this work are summarized in Table 1.

### 2.3. Oligonucleotides

The oligonucleotides used to cloning and mutagenesis are shown in Table 2.

### 2.4. Expression and Purification of Recombinant Proteins

Single colonies of *E. coli* BL21 (*DE3*) strain containing the expression plasmids were inoculated in LB medium (20 mL) containing 0.1 mg ampicillin/mL overnight at 37 °C/250 rpm, transferred to 1 L of fresh LB medium, and cultured further until OD_600_ reached 0.6–0.8. Then the expressions of all proteins were induced by the addition of 0.3 mM IPTG at 37 °C/3 h/250 rpm in orbital shaker. Tsa2 and Tsa2^S44T^ were expressed using similar protocols; however, *E. coli* Tuner (*DE3*) was used instead.

Cells were harvested by centrifugation and pellets were resuspended in start buffer (50 mM sodium phosphate buffer, pH 7.4, 50 mM NaCl, 20 mM imidazole, and 2 mM PMSF) and disrupted by sonication. The cell extracts were kept in ice during streptomycin sulfate 1% treatment for 20 min. The supernatants clarified by centrifugation were homogenized by filtration and purified by IMAC using HisTrap column (GE Healthcare, Piscataway, NJ, USA). Imidazole was removed by gel filtration using PD10 Desalting column (GE Healthcare, Piscataway, NJ, USA) and the purity of recombinant proteins was verified by SDS–PAGE. The yeast Trx1 was purified by boiling method as described previously [53].

### 2.5. NADPH Oxidation Assay

The NADPH to NADP^+^ oxidation was monitored at *A*_340nm_ in 100 μL reaction mixtures containing 50 mM HEPES pH 7.4, 100 μM DTPA, 1 mM sodium azide, 1 μM Prx (Tsa1, Tsa2, or reciprocal mutants), 2 μM Trx1, 0.3 μM TrxR1, and 150 μM NADPH. In some cases, yeast enzymes were replaced by bacterial enzymes (*Pseudomonas aeruginosa* and *Staphylococcus epidermidis* AhpC; *Escherichia coli* Trx and TrxR) at the concentrations described in the legend of the figures. The reactions were initiated by H_2_O_2_ or CHP addition and the reaction mixtures were incubated at 30 °C or 37 °C.

### 2.6. Western Blot Analysis to Evaluate the C_P_ Hyperoxidation

To confirm if 2-Cys Prx were inactivated by C_P_ hyperoxidation, we collected samples after NADPH enzymatic assays. Proteins were pre-alkylated with NEM (50 mM) to avoid the formation of artifactual disulfides before non-reducing SDS-PAGE. Samples were then transferred to a nitrocellulose membrane (Bio-Rad, Hercules, CA, USA) and were incubated with an anti-Prx-SO_3_ (dilution 1:2500; AbFrontier, Seoul, Korea; #LF-PA0004) diluted 1000 times in TBST containing 3% skimmed milk and incubated on a rocking platform for 16 h at 4 °C. A HRP-conjugated secondary antibody (GE Healthcare—Little Chalfont, UK) was employed and samples were detected by chemiluminescence detection (ECL Prime Western Blot, GE Healthcare—Little Chalfont, UK). Images were obtained by ChemiDoc equipment (Bio-Rad—Hercules, CA, EUA).

### 2.7. In Silico Screening of the Typical 2-Cys Prx and Phylogenetic Analyses

The sequences of the *S. cerevisiae* Tsa1 (UniProtKB: P34760), Tsa2 (Q04120), *Salmonella typhimurium* AhpC (P0A251), and *Staphylococcus aureus* AhpC (P0A0B7) were used as eukaryotic and prokaryotic prototypes of typical 2-Cys Prx containing Thr or Ser in the catalytic triad. These amino acid sequences were used as query against TREMBL database using blastp [54] with a cutoff e-value of 10^−3^. Then, to minimize misidentification issues, we identified Pfam domains out of the putative unique orthologs and retained only those presenting the domains found in Tsa1 and Tsa2, AhpC/Prx1 (Pfam: PF00578), redoxin (PF08534) and 1-Cys Prx_C (PF10417) with hmmscan (v.3.1b2) [55]. Sequences presenting other domains, except for SCO1-SenC (PF026300), which we observed in the *S. aureus* AhpC, were excluded. Additionally, because Prx superfamily presents a conserved active-site cysteine P-X-X-X-(T/S)-X-V-C-P-T-E motif [56], known as C_P_-loop [20], we removed sequences lacking this motif. After these filtering steps, we retrieved taxonomic information of the accessions we obtained from the NCBI taxonomy database (database resources of the National Center for Biotechnology Information; June 2018).

### 2.8. Size Exclusion Chromatography (SEC) of Bacterial Typical 2-Cys Prx

To obtain reduced samples, 30 μM of bacterial 2-Cys Prx were treated with TCEP 5 mM for 60 min and the TCEP excess was removed using the PD 10 column (reduced samples). To oxidize samples, reduced 2-Cys Prx (30 μM) were treated with H_2_O_2_ (36 μM) for 30 min, at room temperature. SEC was performed in an analytical Jasco-HPLC system composed by a PU 2880 Plus injector and a PDA MD 2018 detector. Samples (30 μM in 100 mM Tris-HCl at pH 7.4) were separated by a Phenomenex BioSep-SEC-S2000 column (7.8 × 300 mm, 5 μm, resolution range of 15 to 2000 kDa) (Phenomenex, Inc., Torrance, CA, USA), using a flow of 1 mL/min in 100 mM Tris-HCl buffer pH 7.4 monitored by absorbance (280 nm). Bovine thyroglobulin (670 kDa), bovine gamma globulin (158 kDa), ovalbumin (44 kDa), myoglobin (17 kDa) and vitamin B_12_ (1.35 kDa) were used as molecular standards (Bio-Rad Laboratories, Richmond, VA, USA).

### 2.9. Differential Scanning Fluorimetry

The thermal unfolding of bacterial 2-Cys Prx were analyzed by using differential scanning fluorimetry (DSF) [57]. Briefly, proteins (final concentration 3 μM in 10 mM HEPES pH 7.5, 150 mM NaCl) were mixed with 5.25 μL of SYPRO Orange dye diluted 200 times in DMSO. Reactions mixtures were equilibrated at 25 °C for 30 s and then monitored from 25–95 °C with increments of 1 °C/min for 240 min using an Mx 3005P Real-Time System (Agilent Technologies, Santa Clara, CA, USA,) with excitation and emission filters set to 465 and 590 nm, respectively. The melting temperature (*T*_m_) of AhpCs were obtained with the MxPro software and curves fit in GraphPad Prism (GraphPad Software, San Diego, CA, USA) using the Boltzmann equation to determine the midpoint of thermal denaturation (*T*_m_). All measurements were carried out in triplicate and presented as mean ± SD.

### 2.10. Chaperone Activity Assay

Chaperone (holdase) activities of bacterial 2-Cys Prx were analyzed by their ability to inhibit thermally induced aggregation of citrate synthase (CS) as previously described [34]. Briefly, CS (2 μM) was mixed with AhpC at molar ratio 20:1 in 40 mM HEPES-KOH pH 7.5. We also evaluated the differences in chaperone ability of enzymes that were previously heated (48 °C for 1 h). As negative control, samples without the 2-Cys Prx were used. CS aggregation was monitored by light scattering at 360 nm using a Synergy H1 microplate reader (BioTek, Winooski, VT, USA). The assay was executed at 43 °C, with continuously shaking (fast double orbital), with reads at each 15 s for 90 min.

### 2.11. Analysis of Crystallographic Structures

The Prx quaternary structures were accessed from the protein structure database (www.rcsb.org accessed on 5 May 2021). The molecular 3D representations were generated using the PyMOL tool (www.pymol.org accessed on 5 May 2021).

## 3. Results

### 3.1. Presence of a Ser in the Catalytic Triad Increased Resistance of Yeast 2-Cys Prx to Hyperoxidation

Previously, we observed that the presence of a Ser residue in the catalytic triad stabilized yeast 2-Cys Prx in the decameric state even in the disulfide form [3]. Here, we investigated if the presence of a Ser or Thr in the catalytic triad might also affect the hyperoxidation susceptibilities of these enzymes.

**Figure 2:**
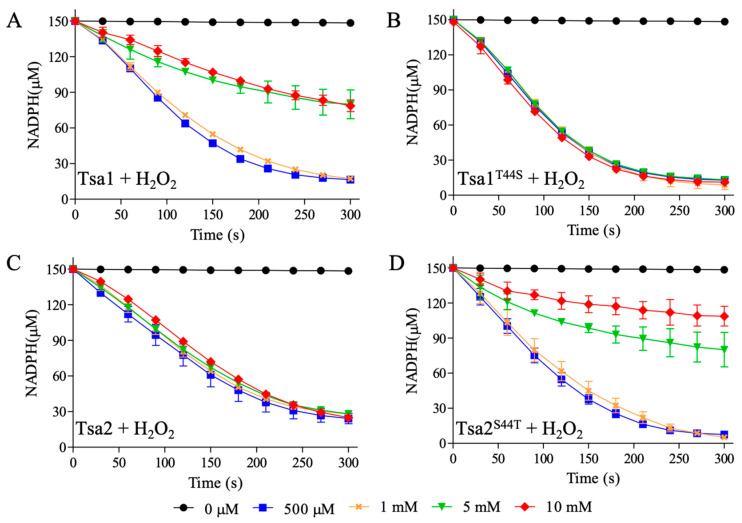
Tsa1, Tsa2, and mutant inhibition by H_2_O_2_. *S. cerevisiae* Tsa1 (**A**), Tsa1^T44S^ (**B**), Tsa2 (**C**), or Tsa2^S44T^ (**D**) were exposed to increasing amounts of H_2_O_2_. The assays were performed with 1 µM Prx, 2 µM Trx1, 0.3 µM TrxR1, and increased amounts of H_2_O_2_ (0 µM = ● black, 500 µM = ■ blue, 1 mM = × orange, 5 mM = ▼ green and 10 mM = ♦ red) in buffer containing 50 mM HEPES pH 7.4, 100 μM DTPA, 1 mM sodium azide and 150 µM NADPH. The decrease in NADPH absorbance was followed at 340 nm. The error bars represent the standard deviation. All the experiments were performed in triplicate and repeated at least three times with similar results.

**Figure 3:**
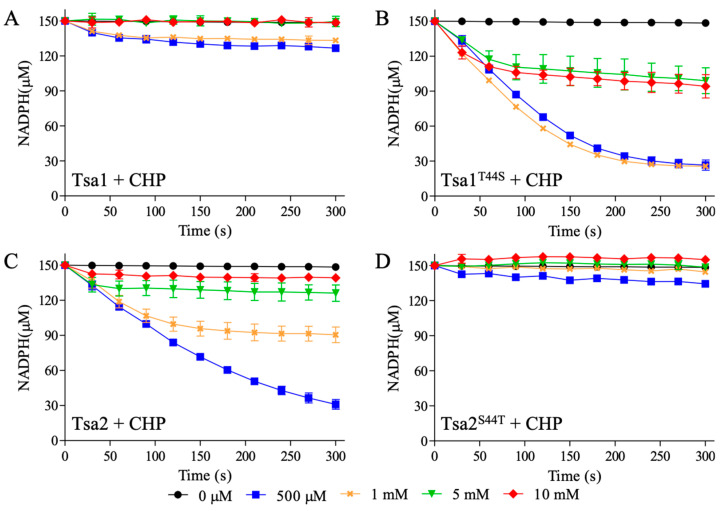
Inactivation of yeast Tsa1, Tsa2, and mutants by CHP. Similar to Figure 2, *S. cerevisiae* Tsa1 (**A**), Tsa1^T44S^ (**B**), Tsa2 (**C**), or Tsa2^S44T^ (**D**) were treated with growing amounts of CHP and the absorbance of NADPH was monitored at 340 nm. The experiment was executed with 1 µM Prx, 2 µM Trx1, 0.3 µM TrxR1 and increased amounts of CHP (0 µM = ● black, 500 µM = ■ blue, 1 mM = × orange, 5 mM = ▼ green and 10 mM = ♦ red) in buffer containing 50 mM HEPES pH 7.4, 100 μM DTPA, 1 mM sodium azide, and 150 µM NADPH. Error bars represent the standard deviations. All the experiments were performed in triplicate and repeated at least three times with similar results.

The decrease of NADPH consumption at increasing hydroperoxides concentrations (H_2_O_2_ in Figure 2 and CHP in Figure 3) is consistent with 2-Cys Prx inhibition by C_P_ hyperoxidation. Tsa1 (a Thr-Prx) was remarkably more susceptible to inactivation (Figure 2A) when compared to Tsa2 (a Ser-Prx) (Figure 2C). At concentrations higher than 1 mM, H_2_O_2_ significantly inhibited Tsa1 (Figure 2A), while high doses of H_2_O_2_ (10 mM) did not inactivate Tsa2 (Figure 2C). Then, NADPH assays with mutant proteins (Tsa1^T44S^ and Tsa2^S44T^) were performed. Tsa1^T44S^ (Figure 2B) was more resistant to H_2_O_2_ inactivation than wild type Tsa1 (Figure 2A). In contrast, Tsa2^S44T^ (Figure 2D) was more susceptible to inactivation than wild type Tsa2 (Figure 2C). Therefore, these results indicated that Thr-Prx are more susceptible to hyperoxidation than Ser-Prx.

We then verified the susceptibilities of these yeast 2-Cys Prx to hyperoxidation by an organic hydroperoxide (CHP). The peroxidase activities of Thr-Prx were almost abolished in all concentrations tested for Tsa1 (Figure 3A) and Tsa2^S44T^ (Figure 3D). In contrast, Ser-Prx were considerably more resistant to inactivation (Figure 3B,C).

We confirmed that 2-Cys Prx inactivation occurred due to C_P_ hyperoxidation, by western blot assays using anti-SO_3_H antibody (Supplementary Material, Figure S1) from aliquots of samples described in Figure 2 and Figure 3. Hyperoxidized 2-Cys Prx migrated as a dimer (~46 kDa) in non-reducing SDS-PAGE, when one of the active sites was in the disulfide form and the other with the C_P_ hyperoxidized (C_P_-SO_3_H) or as monomer, when all C_Ps_ were hyperoxidized.

Two aspects deserve to be highlighted from these results. As already observed [58], Tsa1 is more sensitive to hyperoxidation by CHP than to H_2_O_2_ (Figure 2A and Figure 3A). We now observed that the same characteristic for Tsa2 (Figure 2C and Figure 3C). Additionally, we verified that Ser-Prx (Tsa2 and Tsa1^T44S^) are more resistant to hyperoxidation than Thr-Prx (Tsa1 and Tsa2^S44T^) (Figure 2 and Figure 3). Accordingly, Δ*tsa2* yeast strains are more sensitive to organic hydroperoxides than Δ*tsa1* [59].

### 3.2. Thr-Prx Are More Widely Distributed and Ser-Prx Is More Prevalent in Bacteria

**Figure 4:**
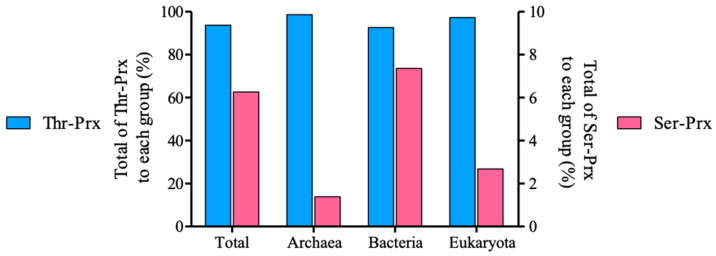
Distribution of the typical 2-Cys Prx containing Thr or Ser as part of the catalytic triad. The analysis of 15,034 unique sequences of typical 2-Cys from the NCBI revealed 14,092 Thr-Prx (93.7%) and 941 Ser-Prx (6.3%) sequences. Considering each domain, Ser-Prx are more abundant at bacteria (7.4%), followed by eukaryotes (2.7%), and archaea (1.4%). The left Y-axis is referent to Thr-Prx, while right Y-axis is related to Ser-Prx.

As the presence of Ser or Thr provokes dramatic alterations in the biochemical and structural properties of yeast 2-Cys Prx, we investigated the phylogenetic distribution of Ser-Prx and Thr-Prx. We analyzed 45,939 unique protein sequences, out of which 15,034 accessions presented AhpC-TSA, Redoxin and 1-CysPrx_C domains denominations according to Pfam and no other domain fused, except for SCO1-SenC. Out of the sequences with taxonomic annotation at domain level, Thr-Prx represented the majority (14,092 sequences, approximately 93.7%), whereas the remaining (941 sequences, approximately 6.3%) are Ser-Prx (Table 3). Notably, the proportions between Thr-Prx and Ser-Prx are different in the three domains of life. Ser-Prx sequences are proportionally more representative in bacteria (7.4%) compared to eukaryotes (2.7%) and archaea (1.4%) (Figure 4). We also found 45 Thr-Prx records deposited in the NCBI database obtained by metagenome approaches and three of them were uncategorized.

The number of sequences of Archaea 2-Cys Prx deposited in the database is very low compared to the other domains of life. Nevertheless, the number of Thr-Prx (71) from Archaea is superior to that of Ser-Prx (1). Thr-Prx are also more widely distributed in Eukarya (2949 representatives present in 12 Superphyla groups) than Ser-Prx (90 sequences found in only four groups) (Table 3). For instance, only one species presents Ser-Prx in chordates (killifish *Fundulus heteroclitus*), whereas a wide range of tetrapods presented only Thr-Prx (See Supplementary Material, Spreadsheet S1).

Concerning bacteria, 16 Superphyla groups contain Thr-Prx, with the prevalence of the Proteobacteria followed by Sphingobacteria (FCB group) and Thermodesulfobacteria groups. Conversely, Ser-Prx occurs with striking predominance in the Terrabacteria group (Table 3). Curiously, Thr-Prx are distributed similarly in Gram negative and Gram positive, whereas Ser-Prx are found mostly in Gram negative (Actinobacteria and Firmicutes). Additionally, as *S. cerevisiae*, some bacteria (e.g., *Bacillus subtilis*) possess both Thr-Prx and Ser-Prx isoforms [60], as *S. cerevisiae*.

### 3.3. Quaternary Structures Characterization of Bacterial Thr-Prx and Ser-Prx

**Figure 5:**
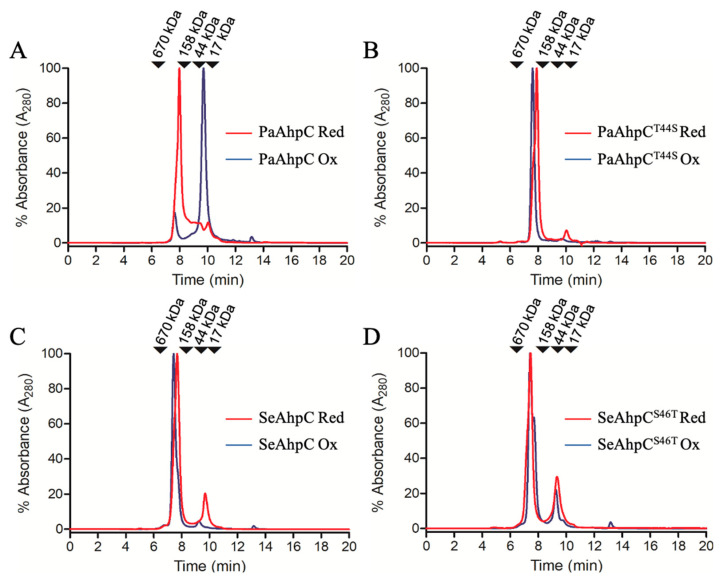
Size exclusion chromatography of PaAhpC and SeAhpC and Thr/Ser mutants. PaAhpC (**A**), PaAhpC^T44S^ (**B**), SeAhpC (**C**), and SeAhpC^S46T^ (**D**), at a final concentration of 30 μM, were treated with TCEP 5 mM (red lines) or H_2_O_2_ 36 μM (blue lines) for 30 min, at room temperature, before being submitted to SEC. The elution profile was monitored at a wavelength of 280 nm, at flow rate of 1 mL/min. Bovine thyroglobulin (670 kDa), bovine gamma globulin (158 kDa), ovalbumin (44 kDa), and myoglobin (17 kDa) were used as molecular standards. The experiments were performed at least three times.

Considering the higher abundance of Ser-Prx in Bacteria, we evaluated the role of Thr/Ser in AhpC enzymes. We selected to analyze recombinant AhpCs from *Pseudomonas aeruginosa* (PaAhpC—a Thr-Prx) and AhpC from *Staphylococcus epidermidis* (SeAhpC—a Ser-Prx), as they belong to the most abundant groups of Thr-Prx (Proteobacteria) and of Ser-Prx (Terrabacteria). As with yeast 2-Cys Prx, we performed reciprocal Ser-Thr mutations and we carried out SEC analysis of bacterial 2-Cys Prx. While PaAhpC was mostly decamer in the reduced form and dimer when oxidized (Figure 5A), PaAhpC^T44S^ was predominantly decamer when oxidized or reduced (Figure 5B). Similarly, yeast Tsa1 and *Salmonella* *typhimurium* AhpC (both Thr-Prx) are decamers when reduced and dimers when oxidized in disulfides, while the substitution of Thr to Ser confers higher stability to the decamer even when the 2-Cys Prx are oxidized [3,39,49].

Similarly to other Ser-Prx [3,48], SeAhpC was presented mostly as decamer independently of the redox state although a small amount of dimers was present in the reduced form (Figure 5C). Additionally, most of SeAhpC^S46T^ was presented as decamer and only a small fraction of the enzyme was detected as dimers, independently of the redox state (Figure 5D). It has been shown by SEC and TEM that the AhpC from *Enterococcus faecalis*, a naturally Ser-Prx, is a decamer independent of the redox state [43], similarly to yeast Tsa2 [3]. Thus, our results showed that the presence of Thr or Ser in the catalytic triad is a relevant factor related to the redox sensitive oligomerization, but additional features also interfere with the quaternary assembly of typical 2-Cys Prx [43,45,61].

### 3.4. Comparative Analysis of the Inactivation of Bacterial Thr-Prx and Ser-Prx by High Hydroperoxide Concentrations and by Thermal Insult

**Figure 6:**
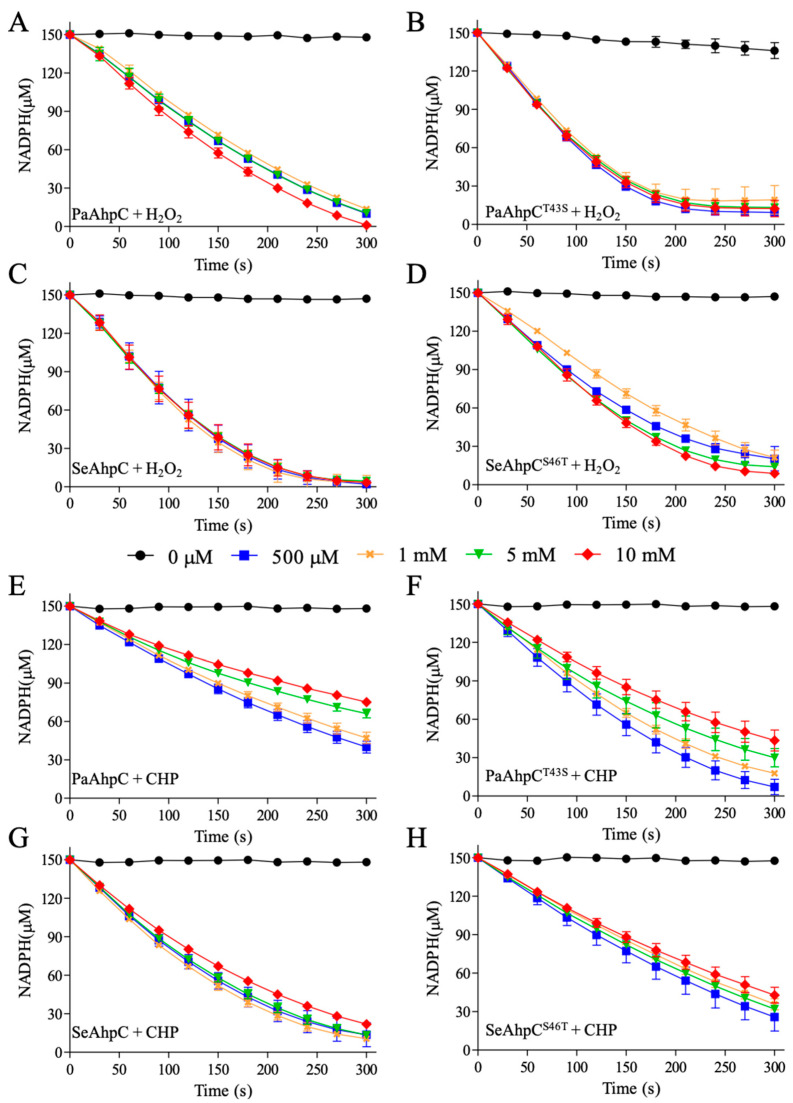
NADPH oxidation assay for evaluation of AhpC hyperoxidation of *P. aeruginosa* and *S. epidermidis* using the heterologous *E. coli* Trx system. Reactions were executed containing PaAhpC (**A**,**E**), PaAhpC^T43S^ (**B**,**F**), SeAhpC (**C**,**G**), or SeAhpC^S46T^ (**D**,**H**) (3 µM), EcTrx (6 µM), EcTrxR (0.9 µM), NADPH (150 µM), 50 mM HEPES pH 7.4, 1 mM sodium azide, and 100 µM DTPA. The experiments were started adding growing concentrations of H_2_O_2_ (**A**–**D**) or CHP (**E**–**H**) (0 µM = ● black, 500 µM = ■ blue, 1 mM = × orange, 5 mM = ▼ green and 10 mM = ♦ red). The reactions were incubated for 5 min/37 °C. The oxidation of NADPH was monitored spectrophotometrically at 340 nm. The error bars represent the standard deviations. All experiments were performed in triplicate and repeated at least three times, yielding consistent results.

We also investigated if the presence of Thr or Ser in the catalytic triad could affect the susceptibility of bacterial 2-Cys Prx to hyperoxidation. In the experimental conditions tested, H_2_O_2_ did not inhibit the NADPH consumption in the reaction mixtures containing Thr-Prx (Figure 6A,D) or Ser-Prx (Figure 6B,C) even at the highest doses (10 mM). However, when CHP was the substrate, a slight inhibition was observed to Thr-Prx (compare Figure 6E,F and Figure 6G,H). Again, we confirmed that the inhibition of peroxidase activity was a consequence of the C_P_ hyperoxidation by western blot analysis performed using anti-SO_3_ antibody (Supplementary Material, Figure S2).

**Figure 7:**
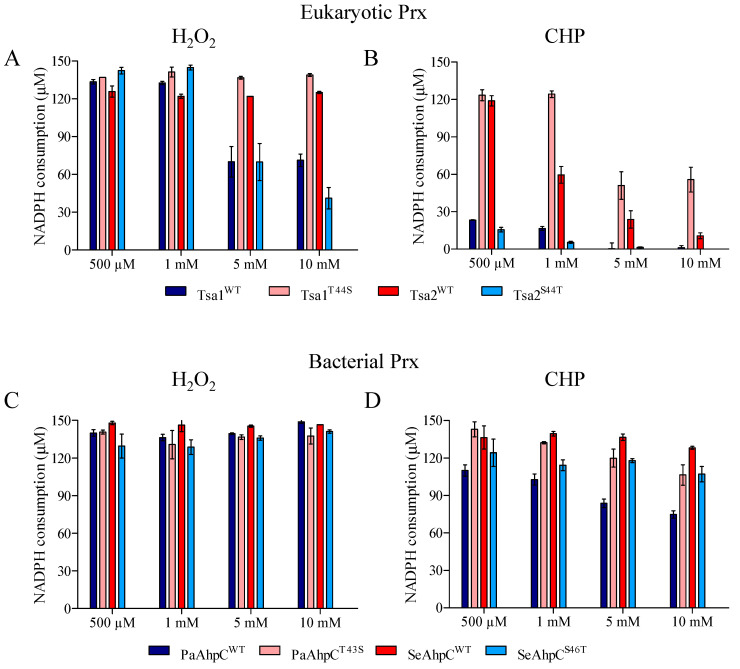
Evaluation of Thr-Prx and Ser-Prx inhibition. The amount of NADPH consumed after 300 s of reaction were obtained in the assays presented in Figure 2, Figure 3, and Figure 6, to H_2_O_2_ (**A**, eukaryotic Prx; **C**, bacterial Prx) and CHP (**B**, eukaryotic Prx; **D**, bacterial Prx). Comparing the values obtained to wild type with the reciprocal enzymes, it is possible to estimate which residue in catalytic triad makes the enzyme more resistant to hyperoxidation. Error bars represent the standard deviations.

As the inhibition to bacterial Thr-Prx was less evident in relation to eukaryotic counterparts, we compared the NADPH consumption after 300 s from the data presented in Figure 2, Figure 3, and Figure 6 (Figure 7). For the yeast enzymes, we observed that NADPH consumption is quite similar between Thr-Prx and Ser-Prx at lower H_2_O_2_ concentrations, but it is considerably higher in samples containing Ser-Prx when 5 or 10 mM H_2_O_2_ were used (Figure 7A). When CHP was the substrate, the NADPH consumption was also more evident for Ser-Prx (Figure 7B). For the bacterial enzymes, there was no clear difference among the enzymes (Figure 7C). However, we could detect a lower NADPH consumption for Thr-Prx (PaAhpC and SeAhpC^S46T^) compared to the reciprocal Ser-Prx (PaAhpC^T43S^ and SeAhpC) (Figure 7D). These results indicate that bacterial Ser-Prx present a subtle resistance to hyperoxidation to CHP, but not to H_2_O_2_ compared to the bacterial Thr-Prx.

**Figure 8:**
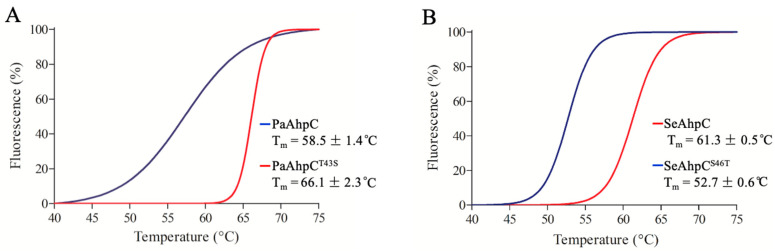
AhpC thermal unfolding monitored by DSF. Differential scanning fluorimetry of PaAhpC (**A**, blue line), PaAhpC^T43S^ (**A**, red line), SeAhpC (**B**, red line), and SeAhpC^S46T^ (**B**, blue line). Proteins were previously reduced with DTT (20 mM), desalted (PD10, GE Healthcare), and incubated with SYPRO Orange dye. The fluorescence scanning was carried out using a temperature gradient from 25 °C to 95 °C. The melting temperature (*T*_m_) was determined by the Boltzmann equation to determine the midpoint of thermal denaturation. The experiments were performed in triplicate and repeated at least three times with similar results.

We also evaluated the inactivation of Thr-Prx and Ser-Prx by thermal stress. The differential scanning fluorimetry (DSF) curve for PaAhpC presented a lag phase and the determined *T*_m_ was 58.5 ± 1.4 °C, while PaAhpC^T44S^ displayed a sudden increase in the fluorescence and the *T*_m_ of 66.1 ± 2.3 °C (Figure 8A). Therefore, replacement of the catalytic triad Thr by Ser resulted in an increase about 8 °C in the *T*_m_ (Figure 8A). For SeAhpC and SeAhpC^S46T^, the shapes of the thermal sigmoidal profiles were very similar, although the Ser to Thr substitution resulted in a decrease of ≈9 °C of the *T*_m_ (61.3 ± 0.5 and 52.7 ± 0.6 °C, respectively) (Figure 8B). Therefore, the presence of Ser in the catalytic triad increased the thermal stabilities of the AhpCs.

**Figure 9:**
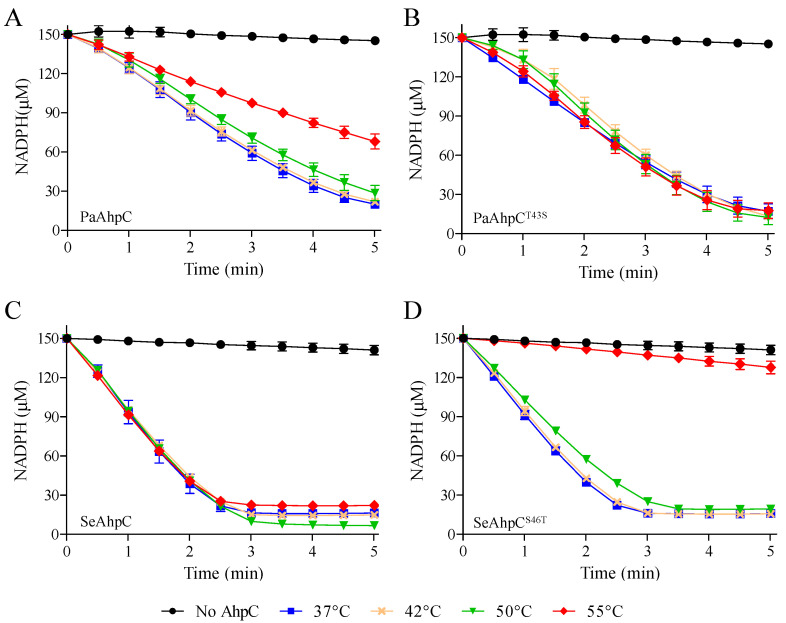
NADPH oxidation assay to assess the effects of thermal inactivation of AhpC enzymes. Aliquots of the PaAhpC (**A**), PaAhpC^T43S^ (**B**), SeAhpC (**C**) and SeAhpC^S46T^ (**D**) were incubated at different temperatures (37 °C = ■ blue, 42 °C = × orange, 50 °C = ▼ green and 55 °C = ♦ red) for 1 h. Then the enzymes were added to the reactions containing 6 µM EcTrx, 0.9 µM EcTrxR, 150 µM NADPH, 50 mM HEPES (pH 7.0), 100 µM DTPA, and 1mM sodium azide. The reactions were initiated by the addition of 500 µM H_2_O_2_ and absorbance at 340 nm was monitored at 37 °C, for 5 min. As negative control (● black), reactions were performed without the addition of the AhpC. The standard deviations are represented by the error bars. All experiments were performed at least three times and yielded similar results using replicates.

Since the thermal stability increased in Ser-Prx, we tested if the proteins retained their peroxidase activities after the thermal pre-treatments. The Thr-Prx (PaAhpC and SeAhpC^S46T^) were partially inactivated after being heated to 55 °C (Figure 9A,D).

However, the effect of temperature was much more noticeable for SeAhpC^S46T^, where the pre-treatment at 55 °C abolished the peroxidase activity of the enzyme (Figure 9D). This result agreed with the determined *T*_m_ values, as the enzyme SeAhpC^S46T^ displayed the lowest *T*_m_ value. On the other hand, the Ser-Prx (PaAhpC^T43S^ and SeAhpC) retained the activity in all temperatures tested (Figure 9B,C).

### 3.5. AhpC Containing Ser Presented Enhanced Chaperone Activity

**Figure 10:**
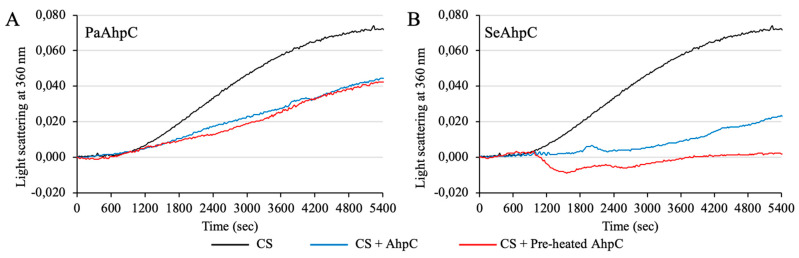
Comparative protection of PaAhpC and SeAhpC to citrate synthase (CS) aggregation by thermal stress. Chaperone activity of PaAhpC and SeAhpC was monitored by its ability to protect citrate synthase (CS) from thermally induced aggregation. The aggregation of 2 μM of CS in 40 mM HEPES-KOH pH 7.5 was monitored by light scattering (λ = 360 nm) for 90 min, either alone or in the presence of 40 μM PaAhpC (**A**) or SeAhpC (**B**). The assays were executed with the enzyme pre-heated at 48 °C for 1 h (red lines) or not (blue lines). The CS alone was used as negative control (black lines). The experiments were performed in triplicates under the same conditions.

We also investigated the abilities of these AhpCs to protect citrate synthase (CS) against thermal insult. CS slowly aggregated when kept at 48 °C as indicated by increased light scattering as a function of time (Figure 10A,B, black lines). In the presence of PaAhpC, the CS aggregation decreased significantly (Figure 10A, blue line). We also tested if the pre-treatment of PaAhpC at 48 °C by 1 h was able to exert additional protection to CS, but no difference was observed in comparison to the untreated enzyme (Figure 10A, red line). In the case of SeAhpC, the sample without thermal pre-treatment presented a more pronounced protection activity (Figure 10B, blue line) than PaAhpC. Notably, pre-heated SeAhpC (48 °C/1 h) protected CS with very high efficiency, because only a negligible amount of CS aggregated (Figure 10B, red line). Therefore, our results suggest that AhpCs containing Ser in the catalytic triad are more prone to act as chaperone induced by heat shock stress than their counterparts containing Thr.

The ability of the typical 2-Cys Prx to act as chaperone (holdase) was already described for several enzymes from eukaryotes [32,33] and from some bacterial proteins [34,62,63]. These enzymes are able to switch from peroxidase to chaperone in response to oxidative stress, by hyperoxidation of C_P_ or by thermal stress. Considering the high resistance of AhpC to C_P_ hyperoxidation, the thermal stress may be the main factor that triggers the chaperone activity, as observed by Kamariah and coworkers (2018) using *E. coli* AhpC (Thr-Prx) as model [34]. Here, we observed the same effect for SeAhpC, which is a Ser-Prx. It is tempting to speculate that chaperone activity may be facilitated in these enzymes because they are stabilized as decamers, irrespective to the oxidative state.

## 4. Discussion

The roles of Thr or Ser in the catalytic triad of the typical 2-Cys Prx has been considered redundant, but recently, we (and others) showed that Ser-Prx are more stable in the decameric form [3,49]. Here, we demonstrated that the presence of Ser or Thr in the active site affected also the C_P_ hyperoxidation susceptibilities, especially for yeast 2-Cys Prx, besides being related to thermal resistance and the chaperone activities acquisition of AhpCs. Furthermore, we showed that the vast majority of multicellular organisms have Thr on catalytic triad. Ser-Prx is more prevalent in bacteria, which might be related with diverse ecological strategies in some groups of this domain. Although the typical 2-Cys Prx from bacteria are very resistant to hyperoxidation, we demonstrated that the Thr to Ser substitution slightly increased the resistance to inactivation of AhpC by CHP.

Previously, eukaryotic 2-Cys Prx were described to be highly susceptible to oxidative inactivation in comparison with bacterial counterparts [40]. Accordingly, eukaryotic 2-Cys Prx were named as sensitive, while bacterial enzymes were referred as robust. Furthermore, authors identified the presence of GGLG and YF motifs exclusively in eukaryotic 2-Cys Prx, which were proposed to be positively selected during evolution [40]. The presence of the GGLG and YF motifs near the C_P_ residue would represent a steric hindrance effect, decreasing the rate of intermolecular disulfide bond formation [37]. According to this model, C_R_ and H_2_O_2_ compete for C_P_-SOH. Therefore, enzymes with fast rates of condensation reaction between C_R_ and C_P_-SOH would correspond to increased resistance to hyperoxidation. More recently, two other structural elements, named motifs A and B, were identified by conferring resistance to hyperoxidation in 2-Cys Prx [50].

Here, we analyzed the susceptibilities to hyperoxidation of sensitive (Tsa1, Tsa2) and robust (PaAhpC, SeAhpC) 2-Cys Prx carrying Thr or Ser in the catalytic triad. Remarkably, yeast Ser-Prx (Tsa1^T44S^ and Tsa2) are considerably more resistant to inactivation than Thr-Prx (Tsa1 and Tsa2 ^S44T^) by hyperoxidation (Figure 2, Figure 3, and Figure 7) and this effect is only moderate in bacterial enzymes treated with CHP (Figure 6 and Figure 7). Noteworthy, motifs A and B previously described as resistance factors to hyperoxidation [50] are also highly similar among the yeast and bacterial counterparts (Supplementary material, Table S1 and Figure S3). Therefore, the observed differences in inactivation between Tsa1 and Tsa2 or between PaAhpC and SeAhpC are probably not related to the compositions of motifs A and B in these enzymes. In fact, the natural substitution of catalytic triad Thr to Ser stands as a novel trait related to resistance or susceptibility to C_P_ hyperoxidation.

**Figure 11:**
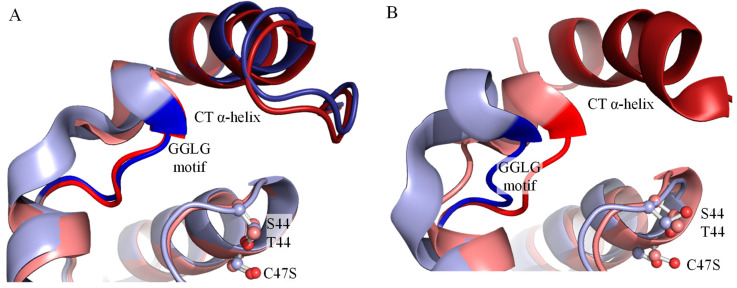
Active site, GGLG and YF motif regions of Tsa1 and Tsa2. (**A**) Superposition of Tsa1^C47S^ (3SBC = pink) and Tsa2^C47S^ (5DVB = light blue) show similar position of Thr^44^ (Tsa1) and Ser^44^ (Tsa2). Motif GGLG (red in Tsa1 and blue in Tsa2) and CT α-helix (dark red in Tsa1 and dark blue in Tsa2) are also in quite similar position. (**B**) When Ser^44^ is found in an alternative conformation, GGLG motif is displaced to a further position, while CT α-helix do not appear in the crystallographic structure probably due to being more mobile and, consequently, with low resolution. Crystallographic structures are presented in cartoon and highlighted amino acids are presented as balls and sticks. Graphical models were generated by PyMOL.

We also analyzed the crystallographic structures of Tsa1 (PDB = 3SBC) and Tsa2 (PDB = 5DVB) to gain insights on mechanisms underlying differences in the hyperoxidation susceptibilities. In our previous work [3], we observed that the side chain of catalytic Ser presents higher mobility than the Thr in Tsa1. The additional methyl group in Thr side chain interacts with an aromatic ring in the adjacent dimer. Thus, the B-factor of active site regions in the 10 chains of Tsa2 structure are higher than observed to Tsa1. Now, we analyzed the interactions of catalytic Thr and Ser in an attempt to explain the differences in hyperoxidation resistance. We observed that the Ser^44^ in some chains of Tsa2 are in position quite similar to Thr^44^ in Tsa1 (Figure 11A). However, in three chains, the Ser^44^ of Tsa2 are found in an alternative position compared to that one observed to Thr^44^. Consequently, the motif GGLG is moved away from the C-terminal α-helix where YF motif is found (Figure 11B) and a helix upstream to the GGLG motif is also deformed. We propose that the high mobility of Ser side chain makes the interaction between GGLG and C-terminal motif YF more unstable and, therefore, facilitate the approximation between C_P_ and C_R_ and disulfide formation. In fact, our data indicated that, in yeast enzymes (displaying the GGLG/YF motifs), the differences in hyperoxidation are more pronounced between Ser-Prx and Thr-Prx. Accordingly, Peskin and colleagues (2021) [61] showed that substitution of human Prx2 C_R_ by amino acids that disrupts the C-terminal structure is able to affect the enzyme activity, indicating an interconnection between the C-terminal region and the active site microenvironment in eukaryotes. These data indicate that even regions located far from the active site can exert effect in the 2-Cys Prx activity.

In contrast, Thr/Ser substitutions in PaAhpC and SeAhpC produce less pronounced effects on the susceptibilities to hyperoxidation, possibly due to GGLG and YF motifs are absent at these enzymes. We compared the crystallographic structure of a Thr-Prx (*Salmonella typhimurium* AhpC-PDB entry 1YEP) with the only AhpC containing Ser in the PDB database (*Enterococcus faecalis*—PDB entry 5Y63), but we did not observe any major structural differences. However, it is worth to mention that disulfide formation in bacterial AhpC belonging to Thr-Prx group is considerable faster (75 s^−1^) compared to human enzymes such as Prx2 and Prx3 (1.7 and 22 s^−1^, respectively) [38,64].

We also observed that 2-Cys Prx are more susceptible to hyperoxidation by organic hydroperoxides than H_2_O_2_ (Figure 2, Figure 3 and Figure 6), as previously described [34], which may be related with the environment close to the active site pocket. Crystallographic observations revealed the presence of large hydrophobic portions around this region [40,43,48]. In fact, *ahpc* gene inactivation frequently render bacteria with increased sensitivity to organic peroxide and AhpC over-expression invariably leads to a gain of resistance against these oxidants [65,66,67,68,69,70,71]. Moreover, *Mycobacterium tuberculosis* and *Helicobacter pylori* mutants for *ahpc* gene accumulated higher levels of lipid hydroperoxides during aerobic growth, when compared to wild type strains [72].

Considering that presence of Ser in catalytic triad makes 2-Cys Prx resistant to C_P_ hyperoxidation, more stable as a decamer and increase its chaperone activity, we speculate that these enzymes may present complementary functions, protecting cells from severe stressful conditions. Ser-Prx might be more prone to hydroperoxides detoxification and thermal stress defense, while Thr-Prx would be better adapted to cell signaling functions. Notably, Ser-Prx were able to perform both peroxidase and chaperone activities after heat shock and possibly these molecular activities may occur simultaneously. Consistent with this idea, Ser-Prx are more frequent in bacteria, some of them pathogenic. Therefore, Ser-Prx might be considered as target for the development of specific inhibitors to prokaryotic enzymes. Finally, it is amazing to note that a single residue substitution by another with similar properties can confer remarkable functional and structural differences between very homologous enzymes.

## 5. Conclusions

The single natural substitution of the active site catalytic triad Thr to Ser occurs in several 2-Cys Prx from different organisms and influences the oligomeric redox state, resistance to hyperoxidation, thermostability, and chaperone activity defining two groups among the 2-Cys Prx: Ser-Prx and Thr-Prx. This substitution may have been selected during the evolutive process and can be directly related to differential functions of these enzymes in the cells.

## Figures and Tables

**Table 1:** List of strains used in this work.

Species	Strain/Plasmid	Source
*E. coli*	ATCC25922	ATCC *
*E. coli*	BL21 DE3/pET15b-*ahpc* (Pa)	This work
*E. coli*	BL21 DE3/pET15b-*ahpc* (Se)	This work
*E. coli*	BL21 DE3/pET15b-*trx* (Ec)	This work
*E. coli*	BL21 DE3/pET15b-*trxr* (Ec)	This work
*E. coli*	BL21 DE3/pET15b-*tsa1* (Sc)	[52]
*E. coli*	BL21 DE3 Tuner/pET15b-*tsa2* (Sc)	[3]
*E. coli*	BL21 DE3/pET15b-*tsa1t44s* (Sc)	[3]
*E. coli*	BL21 DE3 Tuner/pET15b-*tsa2s44t* (Sc)	This work
*E. coli*	BL21 DE3/pET15b-*trx1* (Sc)	[51]
*E. coli*	BL21 DE3/pET15b-*trxr1* (Sc)	[51]
*E. coli*	XL1-Blue	Agilent
*P. aeruginosa*	ATCC29853	ATCC *
*S. epidermidis*	ATCC12228	ATCC *

* American Type Culture Collection; genes: Ec = *E. coli*, Pa = *P. aeruginosa*, Sc = *S. cerevisiae* and Se = *Staphylococcus epidermidis*.

**Table 2:** The oligonucleotides sequences were defined based on NCBI genomic DNA sequences NC_000913.3 (*E. coli ahpc*, *trx* and *trxr*), NC_002516.2 (*P. aeruginosa ahpc*), NZ_CP035288.1 (*S. epidermidis ahpc)* and NC_001136.10 (*S. cerevisiae tsa2*).

Oligonucleotide	Sequence
Ec_Trx_F	5′ CGC GAT CCA TAT GAT GAG CGA TAA AAT TAT TCA CCT GAC T 3′
Ec_Trx_R	5′ CGC AAG CTT GGA TCC TTA CGC CAG GTT AGC GTC GAG GAA 3′
Ec_TrxR_F	5′ CGC GAT CCA TAT GAT GGG CAC GAC CAA ACA CAG TAA ACT G 3′
Ec_TrxR_R	5′ CGC AAG CTT GGA TCC TTA TTT TGC GTC AGC TAA ACC ATC 3′
Pa_AhpC_ F	5′ CGC GAT CCA TAT GAT GTC CCT GAT CAA CAC TCA AGT CCA A 3′
Pa_AhpC_R	5′ CGC AAG CTT GGA TCC TTA GAT CTT GCC GAC CAG GTC CAG G 3′
Pa_AhpC_T43S_F	5′ GCT GCC TTC TCC TTC AAC TGC 3′
Pa_AhpC_T43S_R	5′ GCA GTT GAA GGA GAA GGC AGC 3′
Se_AhpC_S46T_F	5′ GCG GAC TTC ACC TTT GTT TGC 3′
Se_AhpC_S46T_R	5′ GCA AAC AAA GGT GAA GTC CGC 3′
Tsa2S44T_F	5′ ATT GGC TTT TAC TTT TGT CTG TC 3′
Tsa2S44T_R	5′ GAC AGA CAA AAG TAA AAG CCA AT 3′

* Underlined bases indicate *Nde* I and *Bam* HI cloning adapters.

**Table 3:** Distribution of Thr-Prx or Ser-Prx among the living groups.

Thr				Ser			
Bacteria		Eukarya		Bacteria		Eukarya	
Acidobacteria	15	Alveolata	107	FCB group *	14	Amoebozoa	1
Aquificae	16	Amoebozoa	83	Proteobacteria	15	Euglenozoa	12
Calditrichaeota	2	Apusozoa	2	PVC group ^§^	12	Opisthokonta	75
Chrysiogenetes	1	Cryptophyta	1	Spirochaetes	2	Rhodophyta	2
Deferribacteres	5	Euglenozoa	105	Synergistetes	1		
Elusimicrobia	18	Heterolobosea	1	Terrabacteria group	803		
Environmental samples	3	Opisthokonta	2079	Unclassified	4		
FCB group *	1455	Parabasalia (parabasalids)	13				
Fusobacteria	78	Rhizaria	4				
Nitrospinae/Tectomicrobia group	11	Rhodophyta	116				
Nitrospirae	15	Stramenopiles	136				
Proteobacteria	4890	Viridiplantae	302				
PVC group	220						
Spirochaetes	149						
Synergistetes	10						
Terrabacteria group	3564						
Thermodesulfobacteria	2						
Unclassified	245						

* Superphylum that comprises Fibrobacteres, Chlorobi, and Bacteroidetes phyla, ^§^ Superphylum that comprises Planctomycetes, Verrucomicrobia, and Chlamydiae phyla.

## Data Availability

The sequence of the *S. epidermidis ahpc* synthetic gene with the codons optimized for expression in *E. coli* is available at NCBI GenBank (accession code MZ133620).

## Supplementary Materials

The following are available online at https://www.mdpi.com/article/10.3390/antiox10071032/s1, Figure S1: Western blot to confirm C_P_ hyperoxidation of Tsa1 and Tsa2; Figure S2: Evaluation of AhpC hyperoxidation by western blotting; Figure S3: Sequence alignment of Tsa1, Tsa2 and bacterial typical 2-Cys Prx to evaluate the conservation of the hyperoxidation resistance motifs (named A and B) and catalytic triad Thr or Ser distribution; Spreadsheet S1: Distribution of typical 2-Cys Prx among the organisms; Table S1: Conservation of hyperoxidation resistance motifs in typical 2-Cys peroxiredoxins isoforms.
