# Supplementary material for: Effects of Serine or Threonine in the Active Site of Typical 2-Cys Prx on Hyperoxidation Susceptibility and on Chaperone Activity
Source: Antioxidants (Basel). 2021 Jun 25;10(7):1032. doi: 10.3390/antiox10071032 (PMC8300647; doi:10.3390/antiox10071032)
Supplement: Supplementary file 1 [file antioxidants-10-01032-s001.zip › antioxidants-1229985 - supplementary/antioxidants-1229985 - supplementary.pdf]

Effects of Serine or Threonine in the Active Site of Typical 2-Cys Prx on Hyperoxidation Susceptibility and on Chaperone Activity

Supplementary Material

Carlos A. Tairum Jr[1,2§], Melina Cardoso Santos[1§], Carlos Alexandre Breyer[1§], Ana Laura Pires de Oliveira[1], Vitoria Isabela Montanhero Cabrera[1], Guilherme Toledo-Silva[3], Gustavo Maruyama Mori[4], Marcos Hikari Toyama[1], Luis Eduardo Soares Netto[2] and Marcos Antonio de Oliveira[1*]

1 Instituto de Biociências, Universidade Estadual Paulista, UNESP, São Vicente, SP, Brazil.
2 Departamento de Genética e Biologia Evolutiva, Instituto de Biociências, Universidade de São Paulo, São Paulo, SP, Brazil.
3 Laboratório de Biomarcadores de Contaminação Aquática e Imunoquímica, Departamento de Bioquímica, Universidade Federal de Santa Catarina, Florianópolis, SC, Brazil.
4 Laboratório de Ecologia Molecular, Instituto de Biociências, Universidade Estadual Paulista, UNESP, São Vicente, SP, Brazil.

[§] These authors contributed equally to this work.

[*] Correspondence: Marcos A. de Oliveira – marcos.a.oliveira@unesp.br; Luis E.S. Netto – nettoles@ib.usp.br

Table S1. Conservation of hyperoxidation resistance motifs in typical 2-Cys peroxiredoxins isoforms. The hyperoxidation resistance motifs in enzymes containing Thr or Ser in catalytic triad from bacteria, yeast and human. In gray lines are highlighted the enzymes used in this work.

Protein	Motif A region D(N/G)H(S/G)	Motif B region T(S/T)
AhpC_C.j. (Thr)	KGEA	TA
AhpC_P.a. (Thr)	NGHG	TT
AhpC_S.t. (Thr)	DGHG	TT
AhpC_Y.p. (Thr)	HGEA	KQ
AhpC_B.a. (Ser)	DGQA	TA
AhpC_B.s. (Ser)	EGHG	SS
AhpC_E.f. (Ser)	ENHA	NA
AhpC_S.e. (Ser)	NGHG	ST
Tsa1_S.c (Thr)	EGEA	NS
Tsa2_S.c (Ser)	DGEA	NS
Prx1_H.s. (Thr)	DNHS	KA
Prx2_H.s. (Thr)	NGQA	TS
Prx3_H.s. (Thr)	DNHS	TS
Prx4_H.s. (Thr)	DNQS	TS

Abbreviations and Uniprot code: AhpC_C.j.= *Campylobacter jejuni* (Q0PBH5); AhpC_P.a. = *P. aeruginosa* (Q02UU0T); AhpC_S.t. = *Salmonella typhimurium* (P0A251); AhpC_Y.p. = *Yersinia pestis* (Q0WC89); AhpC_B.c. = *Bacillus cereus var. anthracis* (D8GYV3); AhpC_B.s. = *Bacillus subtilis* (P80239); AhpC_E.f. = *Enterococcus faecalis* (O30738) AhpC_S.e. = *S. epidermidis* (Q8CMQ2); Tsa1_S.c. = *S. cerevisiae* (P34760); Tsa2_S.c. = *S. cerevisiae* (Q04120); Prx1_H.s. = *Homo sapiens* (Q06830); Prx2_H.s. = *H. sapiens* (P32119); Prx3_H.s. = *H. sapiens* (P30048) and Prx4_H.s. = *H. sapiens* (Q13162).

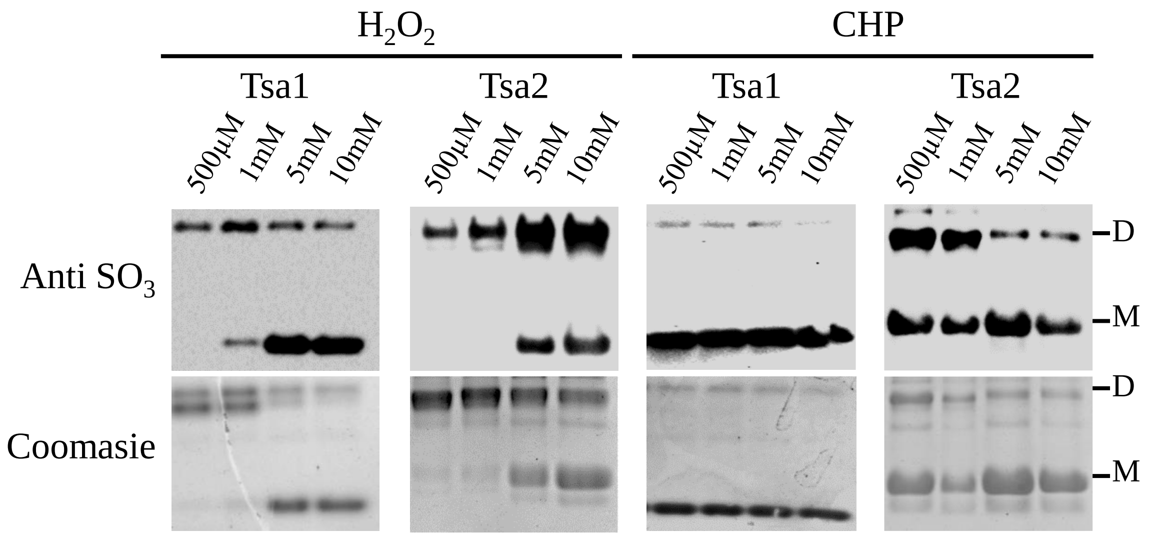

Figure S1. Western blot to confirm C_P hyperoxidation of Tsa1 and Tsa2. The anti- SO_3 antibody were used to verify if Tsa1 and Tsa2 were being hyperoxidized after NADPH assay with growing concentrations of H_2O_2 or CHP (500 µM, 1 mM, 5 mM and 10 mM) (upper panels). SDS-PAGE colored by Coomassie blue are presented in lower panel as loading control. The legends at the right side of the figures are: M = monomer and D = dimer.

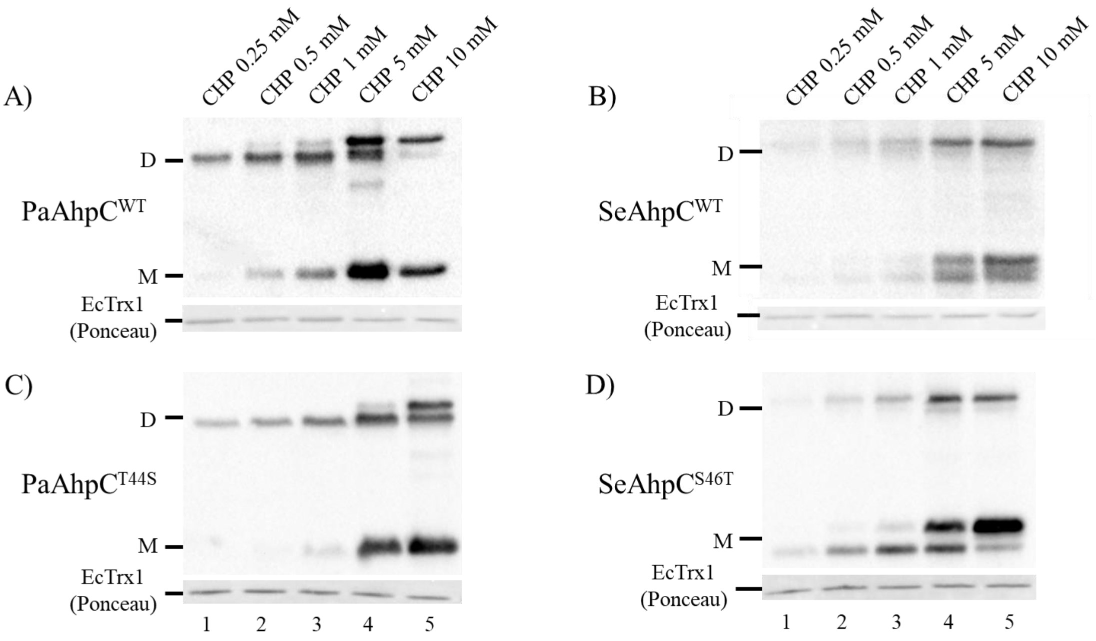

Figure S2. Evaluation of AhpC hyperoxidation by western blotting. The results of the experiments were carried out in the presence of PaAhpC (A), SeAhpC (B), PaAhpC^{T44S} (C) or SeAhpC^{S46T} (D) (3 μM), EcTrx1 (6 μM), EcTrxR (0.9 μM), NADPH (1 mM), sodium azide (100 μM) and increasing concentrations of CHP (0.25 mM, 0.5 mM, 1 mM, 5 mM and 10 mM, lanes 1-5, respectively) treated for 10 minutes at 37°C. The membranes were incubated with the anti-PRDX-SO_3 polyclonal primary antibody (AbFrontier) for 2 hours at room temperature and revealed using the ChemiDoc™ MP Imaging System photodocumentator (Bio-Rad). D = dimers; M = monomers.

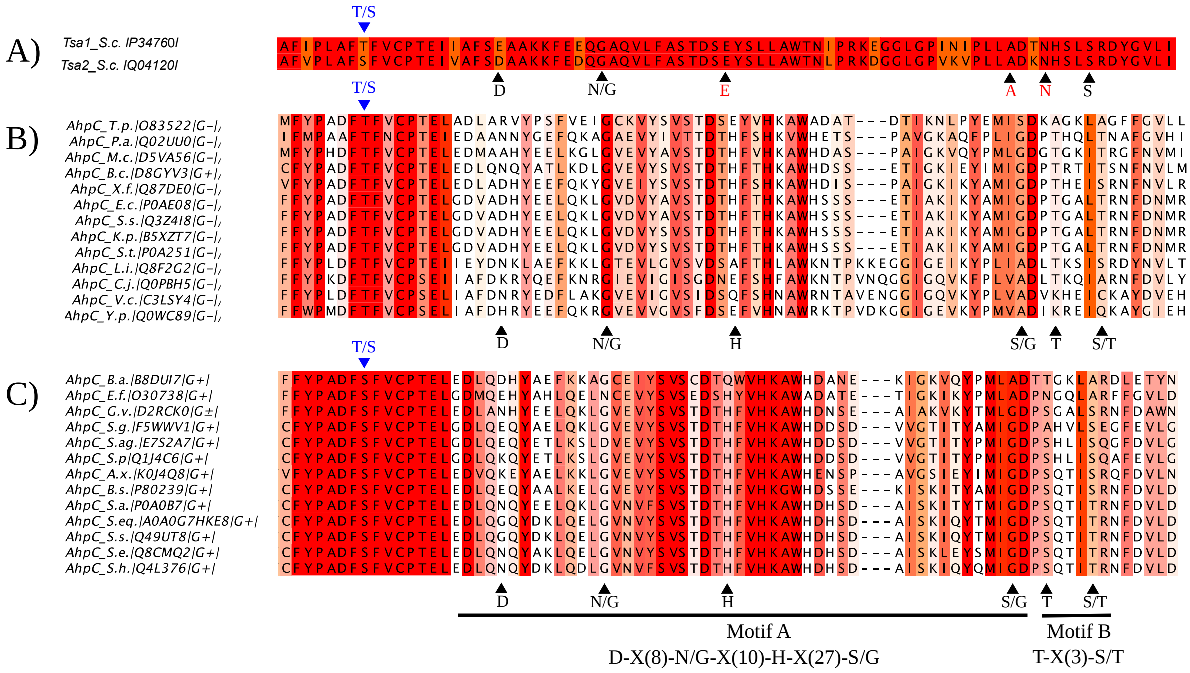

Figure S3. Sequence alignment of Tsa1, Tsa2 and bacterial typical 2-Cys Prx to evaluate the conservation of the hyperoxidation resistance motifs (named A and B) and catalytic triad Thr or Ser distribution. The shading from red to white denotes the degree of amino acid conservation (the redder, the highest the conservation). The blue arrows indicate the presence of Thr or Ser in the catalytic triad. The black arrows indicate the substitutions of amino acids belonging to the motifs A (D-X8-N/G-X10-H-X27-S/G) and B (T-X3-S/T) related to C_p hyperoxidation (Bolduc et al., 2018). **A)** Tsa1 and Tsa2 amino acid alignment (Uniprot access code P34760 and Q04120). **B)** and **C)** alignment of amino acid sequences of bacterial typical 2-Cys Prx containing Thr (**B**) or Ser (**C**) as part of their catalytic triad. The abbreviations of bacterial species used and uniprot access code are as follow: AhpC_T.p. = *Treponema pallidum* (O83522); AhpC_P.a. = *Pseudomonas aeruginosa* (Q02UU0T AhpC_M.c. = *Moraxella catarrhalis* (D5VA56); AhpC_B.c. = *Bacillus cereus* var. anthracis (D8GYV3); AhpC_X.f. = *Xylella fastidiosa* (Q87DE0); AhpC_E.c. = *Escherichia coli* (P0AE08); AhpC_S.s. = *Staphylococcus saprophyticus* (Q49UT8); AhpC_K.p. = *Klebsiella pneumoniae* (B5XZT7); AhpC_S.t. = *Salmonella typhimurium* (P0A251); AhpC_L.i. = *Leptospira interrogans* (Q8F2G2); AhpC_C.j.= *Campylobacter jejuni* (Q0PBH5); AhpC_V.c. = *Vibrio cholerae* (C3LSY4); AhpC_Y.p. = *Yersinia pestis* (Q0WC89); AhpC_B.a. = *Brucella abortus* (Q2YKW3); AhpC_E.f. = *Enterococcus faecalis* (O30738); AhpC_G.v. = *Gardnerella vaginalis* (D2RCK0); AhpC_S.g. = *Streptococcus gallolyticus* (F5WWV1OS); AhpC_S.ag. = *Streptococcus agalactiae* (E7S2A); AhpC_S.p. = *Streptococcus pyogenes* (Q1J4C6); AhpC_A.x. = *Amphibacillus xylanus* (K0J4Q8); AhpC_B.s. = *Bacillus subtilis* (P80239); AhpC_S.a. = *Staphylococcus aureus* (P0A0B7); AhpC_S.eq.= *Streptococcus equi* (A0A0G7HKE8); AhpC_S.s. = *Staphylococcus saprophyticus* (Q49UT8); AhpC_S.e. = *Staphylococcus epidermidis* (Q8CMQ2); AhpC_S.h.= *Staphylococcus haemolyticus* (Q4L376).
